# (+)-Hexacyclinol

**DOI:** 10.1107/S1600536810000620

**Published:** 2010-01-13

**Authors:** David M. Pinkerton, Martin G. Banwell, Anthony C. Willis

**Affiliations:** a Research School of Chemistry, Institute of Advanced Studies, The Australian National University, Canberra, ACT 0200, Australia

## Abstract

A sample of the title compound [systematic name: (1a*S*,2a*S*,3*S*,5a*S*,6a*S*,7*R*,7a*S*,7b*S*,8*R*,8a*S*,10*R*)-7-hydr­oxy-3-(1-meth­oxy-1-methyl­ethyl)-10-(2-methyl-1-propen­yl)-1a,5a,6a,7,7a,7b,8,8a-octa­hydro-2*H*-8,2a-(epoxy­methano)phenanthro[2,3-*b*:6,7-*b′*]bis­oxirene-2,5(3*H*)-dione], C_23_H_28_O_7_, was generated by enanti­oselective synthesis. There are three mol­ecules of the compound in the crystallographic asymmetric unit. Hydrogen bonding between alcohol H atoms and keto groups of adjacent mol­ecules appears to stabilize the structure. The compound is enanti­omerically pure but the absolute configuration could not be determined directly in this study. Accordingly, the illustrated configuration was assigned on the basis of the nature of the chiral nonracemic precursor used in the synthesis.

## Related literature

(+)-Hexacyclinol is a natural product that was first isolated and characterized by Schlegel *et al.* (2002 ▶). Rychnovsky (2006 ▶) proposed the illustrated structure by re-evaluation of the derived ^13^C NMR spectral data. This structure was confirmed through the synthesis and X-ray analysis of racemic material (Porco *et al.*, 2006 ▶). Like others (Mehta & Roy, 2008 ▶), we have recently completed a synthesis of (+)-hexa­cyclinol, in our case from an enzymatically generated and enanti­omerically pure *cis*-1,2-dihydro­catechol (Pinkerton *et al.*, 2009 ▶).
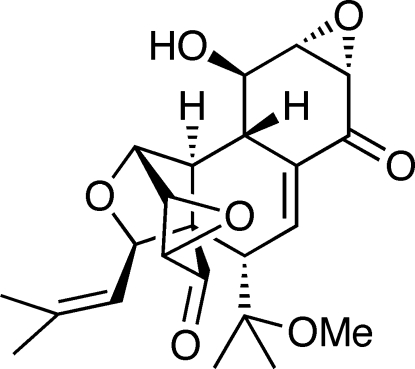


## Experimental

### 

#### Crystal data

C_23_H_28_O_7_
*M* *_r_* = 416.47
Orthorhombic, 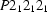

*a* = 10.8270 (1) Å
*b* = 20.0530 (2) Å
*c* = 28.0247 (3) Å
*V* = 6084.55 (11) Å^3^
*Z* = 12
Mo *K*α radiation
μ = 0.10 mm^−1^
*T* = 200 K
0.42 × 0.18 × 0.15 mm

#### Data collection

Nonius KappaCCD diffractometer
Absorption correction: integration [*via* Gaussian method (Coppens, 1970 ▶) implemented in *maXus* (Mackay *et al.*, 2000 ▶)] *T*_min_ = 0.972, *T*_max_ = 0.994
80486 measured reflections
7619 independent reflections
5571 reflections with *I* > 2σ(*I*)
*R*_int_ = 0.036

#### Refinement

*R*[*F* ^2^ > 2σ(*F* ^2^)] = 0.034
*wR*(*F* ^2^) = 0.079
*S* = 0.83
7619 reflections
820 parameters
H atoms treated by a mixture of independent and constrained refinement
Δρ_max_ = 0.54 e Å^−3^
Δρ_min_ = −0.25 e Å^−3^
Absolute structure: the enantiomer has been assigned by reference to an unchanging centre of chirality in the starting material.

### 

Data collection: *COLLECT* (Nonius, 2001 ▶); cell refinement: *DENZO*/*SCALEPACK* (Otwinowski & Minor, 1997 ▶); data reduction: *DENZO*/*SCALEPACK*; program(s) used to solve structure: *SIR92* (Altomare *et al.*, 1994 ▶); program(s) used to refine structure: *CRYSTALS* (Betteridge *et al.*, 2003 ▶); molecular graphics: *ORTEPII* (Johnson, 1976 ▶) in *TEXSAN* (Molecular Structure Corporation, 1997 ▶); software used to prepare material for publication: *CRYSTALS*.

## Supplementary Material

Crystal structure: contains datablocks I, global. DOI: 10.1107/S1600536810000620/hg2629sup1.cif

Structure factors: contains datablocks I. DOI: 10.1107/S1600536810000620/hg2629Isup2.hkl

Additional supplementary materials:  crystallographic information; 3D view; checkCIF report

## Figures and Tables

**Table 1:** Hydrogen-bond geometry (Å, °)

*D*—H⋯*A*	*D*—H	H⋯*A*	*D*⋯*A*	*D*—H⋯*A*
O30—H301⋯O121	0.91 (3)	1.92 (3)	2.828 (3)	172 (3)
O130—H1301⋯O21^i^	0.81 (3)	2.04 (3)	2.796 (3)	155 (3)
O230—H2301⋯O221^ii^	0.76 (3)	2.10 (3)	2.809 (3)	156 (3)

Symmetry codes: (i) 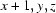; (ii) 
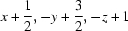
.

**Fig. 1.**
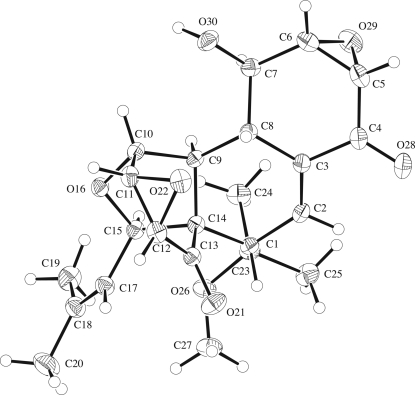
Structure of C23H28O7 molecule one with labelling of selected atoms. Anisotropic displacement ellipsoids display 30% probability levels. Hydrogen atoms are drawn as circles with small radii.

**Fig. 2.**
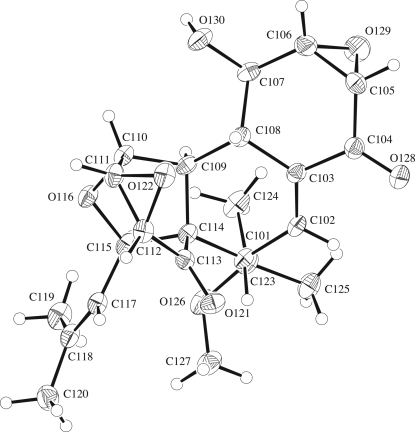
Structure of C23H28O7 molecule two with labelling of selected atoms. Anisotropic displacement ellipsoids display 30% probability levels. Hydrogen atoms are drawn as circles with small radii.

**Fig. 3.**
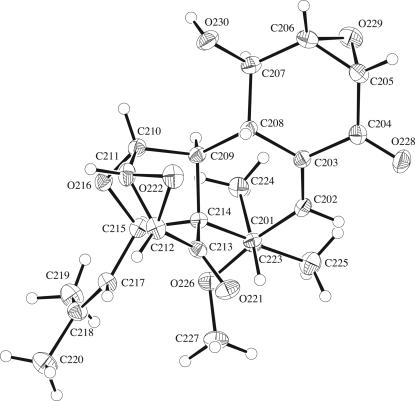
Structure of C23H28O7 molecule three with labelling of selected atoms. Anisotropic displacement ellipsoids display 30% probability levels. Hydrogen atoms are drawn as circles with small radii.

**Fig. 4.**
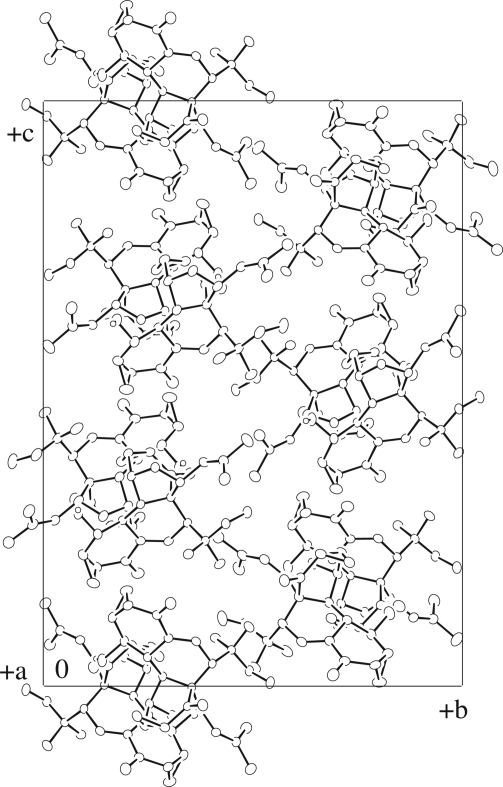
Unit cell packing diagram of C23H28O7 projected down the a axis. Anisotropic displacement ellipsoids display 30% probability levels. The alcohol H atoms are drawn as circles with small radii and the remaining H atoms have been deleted.

## supplementary crystallographic information

### Experimental

(+)-Hexacyclinol was prepared according to a published procedure (Pinkerton *et al.*, 2009) and crystals suitable for X-ray analysis grown from mixtures of dichloromethane and light-petroleum. The sample had m.p. = 176–178°C [lit., Mehta & Roy (2008) m.p. = 179–180°C], [α]_D_ = +129.6 (*c* 0.36, methanol) {lit., Mehta & Roy (2008) [α]_D_ = +130.9 (*c* 0.42, methanol)}.

### Refinement

The compound is enantiometrically pure but the anomolous dispersion terms are very low for all elements in the structure so the absolute configuration could not be determined in this experiment. Consequently, Friedel-pair reflections were averaged and the Flack parameter was not refined. The illustrated configuration was assigned on the basis of the nature of the chiral, non-racemic, precursor used in the synthesis.

H atoms attached to C were included at calculated positions (methyl groups oriented to best-match peaks in a difference electron density map) and those attached to O as indicated in the difference map. The H atoms were initially refined with soft restraints on the bond lengths and angles to regularize their geometry (C—H in the range 0.93–0.98, O—H = 0.82 Å) and with *U*_iso_(H) in the range 1.2–1.5 times *U*_eq_ of the parent atom. Later, H atoms attached to C were refined with riding constraints and those attached to O were allowed to refine without restraints.

The largest peak in the final difference map is located adjacent to C105 but appears to have no chemical significance. Subsequent peaks are located between or adjacent to atoms already located or in the vicinity of methyl Hs.

### Crystal data

**Table:**

C_23_H_28_O_7_	*F*(000) = 2664
*M**_r_* = 416.47	*D*_x_ = 1.364 Mg m^−^^3^
Orthorhombic, *P*2_1_2_1_2_1_	Mo *K*α radiation, λ = 0.71073 Å
Hall symbol: P 2ac 2ab	Cell parameters from 119334 reflections
*a* = 10.8270 (1) Å	θ = 2.6–27.5°
*b* = 20.0530 (2) Å	µ = 0.10 mm^−^^1^
*c* = 28.0247 (3) Å	*T* = 200 K
*V* = 6084.55 (11) Å^3^	Needle, colourless
*Z* = 12	0.42 × 0.18 × 0.15 mm

### Data collection

**Table:**

Nonius KappaCCD diffractometer	5571 reflections with *I* > 2σ(*I*)
graphite	*R*_int_ = 0.036
φ and ω scans with CCD	θ_max_ = 27.5°, θ_min_ = 2.6°
Absorption correction: integration [*via* Gaussian method (Coppens, 1970) implemented in *maXus* (Mackay *et al.*, 2000)]	*h* = −13→13
*T*_min_ = 0.972, *T*_max_ = 0.994	*k* = −26→26
80486 measured reflections	*l* = −25→36
7619 independent reflections	

### Refinement

**Table:**

Refinement on *F*^2^	Primary atom site location: structure-invariant direct methods
Least-squares matrix: full	Hydrogen site location: inferred from neighbouring sites
*R*[*F*^2^ > 2σ(*F*^2^)] = 0.034	H atoms treated by a mixture of independent and constrained refinement
*wR*(*F*^2^) = 0.079	Method = Modified Sheldrick *w* = 1/[σ^2^(*F*^2^) + (0.03*P*)^2^ + 0.0*P*], where *P* = (max(*F*_o_^2^,0) + 2*F*_c_^2^)/3
*S* = 0.83	(Δ/σ)_max_ = 0.001
7619 reflections	Δρ_max_ = 0.54 e Å^−^^3^
820 parameters	Δρ_min_ = −0.25 e Å^−^^3^
0 restraints	Absolute structure: The enantiomer has been assigned by reference to an unchanging centre of chirality in the starting material.

### Fractional atomic coordinates and isotropic or equivalent isotropic displacement parameters (Å^2^)

**Table:**

	*x*	*y*	*z*	*U*_iso_*/*U*_eq_	
C1	0.3484 (2)	0.15601 (11)	0.72085 (8)	0.0299	
C2	0.27315 (19)	0.20115 (12)	0.75220 (8)	0.0320	
C3	0.27084 (19)	0.26780 (12)	0.75158 (8)	0.0309	
C4	0.1877 (2)	0.30240 (13)	0.78622 (8)	0.0369	
C5	0.2149 (2)	0.37427 (13)	0.79603 (9)	0.0448	
C6	0.3238 (2)	0.40522 (13)	0.77555 (9)	0.0426	
C7	0.4057 (2)	0.37003 (11)	0.74015 (9)	0.0339	
C8	0.34130 (18)	0.31058 (11)	0.71650 (8)	0.0276	
C9	0.43535 (19)	0.26764 (10)	0.68923 (8)	0.0272	
C10	0.48673 (19)	0.28963 (11)	0.64103 (8)	0.0309	
C11	0.3911 (2)	0.29732 (12)	0.60224 (9)	0.0358	
C12	0.2960 (2)	0.24587 (12)	0.60020 (9)	0.0370	
C13	0.29373 (19)	0.19483 (12)	0.63865 (8)	0.0309	
C14	0.39485 (19)	0.19549 (11)	0.67712 (8)	0.0281	
C15	0.51777 (19)	0.17370 (11)	0.65151 (8)	0.0309	
O16	0.55958 (14)	0.23319 (7)	0.62627 (6)	0.0337	
C17	0.5156 (2)	0.11678 (12)	0.61741 (8)	0.0357	
C18	0.6034 (2)	0.07004 (13)	0.61481 (9)	0.0412	
C19	0.7156 (2)	0.06746 (14)	0.64595 (10)	0.0504	
C20	0.5942 (3)	0.01329 (15)	0.57995 (12)	0.0664	
O21	0.21643 (15)	0.15080 (9)	0.63683 (7)	0.0481	
O22	0.26572 (15)	0.31279 (9)	0.61589 (6)	0.0419	
C23	0.4397 (2)	0.11377 (11)	0.75171 (8)	0.0347	
C24	0.5434 (2)	0.15375 (12)	0.77433 (9)	0.0418	
C25	0.3664 (3)	0.07883 (13)	0.79109 (9)	0.0466	
O26	0.49970 (15)	0.06476 (8)	0.72235 (6)	0.0393	
C27	0.4243 (3)	0.01340 (12)	0.70271 (10)	0.0493	
O28	0.10381 (16)	0.27442 (10)	0.80699 (6)	0.0515	
O29	0.32731 (18)	0.38064 (10)	0.82386 (6)	0.0555	
O30	0.43866 (17)	0.41634 (9)	0.70388 (7)	0.0461	
C101	0.8589 (2)	0.42906 (12)	0.60656 (8)	0.0341	
C102	0.7833 (2)	0.38365 (12)	0.57556 (9)	0.0353	
C103	0.7806 (2)	0.31715 (12)	0.57742 (8)	0.0337	
C104	0.6989 (2)	0.28108 (13)	0.54294 (9)	0.0397	
C105	0.7219 (2)	0.20815 (13)	0.53624 (9)	0.0447	
C106	0.8344 (2)	0.17845 (14)	0.55588 (9)	0.0475	
C107	0.9195 (2)	0.21771 (12)	0.58843 (9)	0.0393	
C108	0.8517 (2)	0.27511 (12)	0.61317 (8)	0.0320	
C109	0.9392 (2)	0.31835 (11)	0.64262 (8)	0.0333	
C110	0.9816 (2)	0.29827 (12)	0.69246 (8)	0.0349	
C111	0.8784 (2)	0.28925 (13)	0.72856 (9)	0.0397	
C112	0.7785 (2)	0.33811 (13)	0.72586 (9)	0.0384	
C113	0.7795 (2)	0.38726 (12)	0.68501 (9)	0.0336	
C114	0.89379 (19)	0.39013 (11)	0.65232 (8)	0.0317	
C115	1.0048 (2)	0.41569 (12)	0.68319 (9)	0.0353	
O116	1.04747 (14)	0.35658 (8)	0.70936 (6)	0.0385	
C117	0.9802 (2)	0.47235 (11)	0.71650 (8)	0.0354	
C118	1.0530 (2)	0.52478 (13)	0.72361 (9)	0.0421	
C119	1.1778 (2)	0.53375 (14)	0.70100 (11)	0.0518	
C120	1.0139 (3)	0.58253 (13)	0.75441 (10)	0.0538	
O121	0.69216 (14)	0.42477 (8)	0.68032 (6)	0.0430	
O122	0.75780 (15)	0.27021 (9)	0.71160 (6)	0.0420	
C123	0.9601 (2)	0.46485 (13)	0.57641 (9)	0.0413	
C124	1.0661 (2)	0.42028 (14)	0.56013 (10)	0.0518	
C125	0.8989 (2)	0.49591 (14)	0.53231 (10)	0.0501	
O126	1.01865 (15)	0.51608 (9)	0.60449 (7)	0.0487	
C127	0.9463 (3)	0.57282 (13)	0.61580 (12)	0.0642	
O128	0.61975 (17)	0.30835 (9)	0.51944 (7)	0.0567	
O129	0.83095 (19)	0.19847 (11)	0.50687 (7)	0.0645	
O130	0.96384 (17)	0.17434 (10)	0.62467 (8)	0.0522	
C201	0.38514 (18)	0.59697 (10)	0.53867 (8)	0.0272	
C202	0.31476 (19)	0.63543 (11)	0.57649 (8)	0.0306	
C203	0.31062 (18)	0.70143 (11)	0.58224 (8)	0.0289	
C204	0.2323 (2)	0.72855 (12)	0.62163 (8)	0.0340	
C205	0.2576 (2)	0.79795 (12)	0.63791 (9)	0.0381	
C206	0.3626 (2)	0.83442 (13)	0.61786 (9)	0.0408	
C207	0.4391 (2)	0.80679 (11)	0.57711 (9)	0.0350	
C208	0.37384 (19)	0.75120 (10)	0.54964 (8)	0.0285	
C209	0.46373 (19)	0.71508 (10)	0.51667 (8)	0.0284	
C210	0.5095 (2)	0.74653 (11)	0.46994 (8)	0.0331	
C211	0.4117 (2)	0.75913 (12)	0.43313 (9)	0.0387	
C212	0.3113 (2)	0.71052 (12)	0.42960 (9)	0.0365	
C213	0.3068 (2)	0.65565 (11)	0.46596 (8)	0.0299	
C214	0.42090 (19)	0.64566 (10)	0.49810 (8)	0.0263	
C215	0.53408 (19)	0.62933 (11)	0.46475 (8)	0.0300	
O216	0.58483 (13)	0.69366 (7)	0.45007 (6)	0.0342	
C217	0.5121 (2)	0.58804 (11)	0.42100 (8)	0.0330	
C218	0.5857 (2)	0.54003 (12)	0.40475 (9)	0.0361	
C219	0.7006 (2)	0.51586 (14)	0.42860 (10)	0.0494	
C220	0.5579 (3)	0.50506 (13)	0.35848 (9)	0.0530	
O221	0.21737 (14)	0.61965 (8)	0.46774 (6)	0.0431	
O222	0.28873 (15)	0.77640 (8)	0.44880 (6)	0.0423	
C223	0.4851 (2)	0.55123 (11)	0.56191 (8)	0.0312	
C224	0.5945 (2)	0.58940 (12)	0.58251 (9)	0.0363	
C225	0.4271 (2)	0.50934 (13)	0.60141 (9)	0.0441	
O226	0.53916 (14)	0.50852 (7)	0.52626 (6)	0.0361	
C227	0.4617 (3)	0.45880 (12)	0.50607 (10)	0.0473	
O228	0.15206 (15)	0.69639 (9)	0.64143 (7)	0.0491	
O229	0.37478 (17)	0.80146 (10)	0.66304 (6)	0.0531	
O230	0.46259 (17)	0.85956 (8)	0.54406 (7)	0.0469	
H11	0.2880	0.1228	0.7087	0.0348*	
H21	0.2203	0.1799	0.7755	0.0378*	
H51	0.1458	0.4007	0.8105	0.0531*	
H61	0.3250	0.4537	0.7728	0.0501*	
H71	0.4821	0.3547	0.7558	0.0417*	
H81	0.2817	0.3296	0.6934	0.0346*	
H91	0.5089	0.2645	0.7097	0.0320*	
H101	0.5382	0.3306	0.6438	0.0365*	
H111	0.4215	0.3171	0.5723	0.0420*	
H121	0.2599	0.2330	0.5694	0.0445*	
H151	0.5820	0.1641	0.6772	0.0352*	
H171	0.4450	0.1152	0.5943	0.0429*	
H191	0.7870	0.0598	0.6270	0.0764*	
H192	0.7102	0.0297	0.6662	0.0770*	
H193	0.7242	0.1069	0.6652	0.0777*	
H201	0.5970	−0.0286	0.5977	0.1002*	
H202	0.5165	0.0164	0.5626	0.0998*	
H203	0.6631	0.0137	0.5583	0.0997*	
H241	0.5863	0.1254	0.7958	0.0622*	
H242	0.5076	0.1922	0.7912	0.0625*	
H243	0.6007	0.1698	0.7505	0.0621*	
H251	0.4142	0.0428	0.8056	0.0685*	
H252	0.3420	0.1119	0.8148	0.0695*	
H253	0.2894	0.0587	0.7784	0.0695*	
H271	0.4714	−0.0149	0.6817	0.0740*	
H272	0.3852	−0.0151	0.7264	0.0738*	
H273	0.3586	0.0326	0.6832	0.0732*	
H301	0.522 (3)	0.4171 (15)	0.6991 (11)	0.0694*	
H1011	0.8037	0.4647	0.6173	0.0397*	
H1021	0.7336	0.4038	0.5529	0.0415*	
H1051	0.6631	0.1807	0.5153	0.0525*	
H1061	0.8355	0.1286	0.5602	0.0555*	
H1071	0.9860	0.2352	0.5684	0.0469*	
H1081	0.7879	0.2555	0.6352	0.0376*	
H1091	1.0132	0.3239	0.6242	0.0399*	
H1101	1.0372	0.2609	0.6930	0.0400*	
H1111	0.9016	0.2704	0.7596	0.0469*	
H1121	0.7325	0.3514	0.7543	0.0453*	
H1151	1.0702	0.4272	0.6605	0.0407*	
H1171	0.9062	0.4709	0.7336	0.0432*	
H1191	1.1776	0.5717	0.6802	0.0777*	
H1192	1.2374	0.5415	0.7242	0.0772*	
H1193	1.2021	0.4957	0.6829	0.0777*	
H1201	1.0701	0.5917	0.7802	0.0794*	
H1202	0.9308	0.5766	0.7674	0.0800*	
H1203	1.0123	0.6227	0.7368	0.0803*	
H1241	1.1213	0.4445	0.5385	0.0778*	
H1242	1.1125	0.4063	0.5887	0.0768*	
H1243	1.0337	0.3800	0.5451	0.0780*	
H1251	0.9554	0.5308	0.5202	0.0747*	
H1252	0.8186	0.5171	0.5408	0.0741*	
H1253	0.8873	0.4617	0.5091	0.0757*	
H1271	0.9986	0.6034	0.6333	0.0955*	
H1272	0.8811	0.5592	0.6387	0.0951*	
H1273	0.9155	0.5936	0.5878	0.0954*	
H1301	1.036 (3)	0.1665 (17)	0.6191 (12)	0.0785*	
H2011	0.3257	0.5649	0.5260	0.0323*	
H2021	0.2713	0.6104	0.5986	0.0374*	
H2051	0.1885	0.8226	0.6564	0.0451*	
H2061	0.3597	0.8822	0.6186	0.0488*	
H2071	0.5144	0.7894	0.5905	0.0410*	
H2081	0.3092	0.7743	0.5307	0.0334*	
H2091	0.5382	0.7074	0.5353	0.0314*	
H2101	0.5598	0.7862	0.4750	0.0376*	
H2111	0.4356	0.7814	0.4042	0.0460*	
H2121	0.2750	0.7013	0.3983	0.0434*	
H2151	0.5975	0.6077	0.4844	0.0347*	
H2171	0.4429	0.5992	0.4028	0.0392*	
H2191	0.7683	0.5119	0.4054	0.0743*	
H2192	0.7240	0.5450	0.4526	0.0746*	
H2193	0.6840	0.4733	0.4426	0.0751*	
H2201	0.5514	0.4580	0.3637	0.0806*	
H2202	0.6248	0.5136	0.3375	0.0796*	
H2203	0.4798	0.5227	0.3439	0.0796*	
H2241	0.6482	0.5587	0.6004	0.0529*	
H2242	0.5671	0.6226	0.6047	0.0532*	
H2243	0.6414	0.6084	0.5577	0.0540*	
H2251	0.4842	0.4742	0.6093	0.0650*	
H2252	0.4151	0.5360	0.6298	0.0648*	
H2253	0.3499	0.4883	0.5928	0.0657*	
H2271	0.5106	0.4315	0.4843	0.0713*	
H2272	0.4269	0.4310	0.5301	0.0711*	
H2273	0.3944	0.4785	0.4881	0.0715*	
H2301	0.528 (3)	0.8734 (16)	0.5468 (12)	0.0706*	

### Atomic displacement parameters (Å^2^)

**Table:**

	*U*^11^	*U*^22^	*U*^33^	*U*^12^	*U*^13^	*U*^23^
C1	0.0311 (11)	0.0282 (12)	0.0303 (12)	−0.0040 (10)	−0.0020 (10)	0.0032 (10)
C2	0.0276 (11)	0.0381 (13)	0.0302 (12)	−0.0046 (11)	0.0006 (10)	0.0064 (11)
C3	0.0274 (11)	0.0375 (13)	0.0277 (12)	−0.0009 (10)	−0.0022 (9)	0.0006 (11)
C4	0.0325 (12)	0.0472 (15)	0.0310 (13)	0.0042 (12)	0.0020 (11)	0.0011 (12)
C5	0.0453 (14)	0.0513 (16)	0.0377 (14)	0.0101 (13)	0.0035 (12)	−0.0072 (13)
C6	0.0475 (14)	0.0372 (14)	0.0430 (15)	0.0054 (12)	−0.0030 (12)	−0.0067 (12)
C7	0.0276 (11)	0.0317 (13)	0.0422 (14)	0.0014 (10)	−0.0028 (10)	−0.0015 (11)
C8	0.0233 (10)	0.0290 (12)	0.0307 (12)	0.0011 (9)	−0.0020 (9)	−0.0003 (10)
C9	0.0233 (10)	0.0268 (11)	0.0316 (12)	0.0001 (9)	−0.0017 (9)	0.0033 (10)
C10	0.0278 (11)	0.0290 (12)	0.0359 (13)	0.0002 (10)	0.0035 (10)	0.0033 (10)
C11	0.0375 (13)	0.0358 (13)	0.0340 (13)	0.0059 (12)	0.0036 (10)	0.0065 (11)
C12	0.0370 (13)	0.0434 (15)	0.0308 (13)	0.0059 (12)	−0.0055 (11)	0.0023 (11)
C13	0.0255 (11)	0.0345 (13)	0.0328 (13)	0.0022 (11)	−0.0009 (10)	−0.0023 (11)
C14	0.0242 (10)	0.0297 (12)	0.0305 (12)	0.0026 (10)	−0.0016 (9)	0.0014 (10)
C15	0.0274 (11)	0.0313 (12)	0.0339 (13)	0.0030 (10)	0.0007 (10)	0.0017 (10)
O16	0.0312 (8)	0.0304 (8)	0.0395 (9)	0.0032 (7)	0.0061 (7)	0.0005 (7)
C17	0.0344 (12)	0.0382 (13)	0.0344 (13)	0.0009 (12)	−0.0013 (10)	−0.0035 (11)
C18	0.0430 (14)	0.0389 (14)	0.0417 (15)	0.0037 (12)	0.0038 (12)	−0.0015 (12)
C19	0.0373 (13)	0.0491 (16)	0.0649 (19)	0.0092 (13)	0.0010 (13)	0.0056 (15)
C20	0.073 (2)	0.0555 (18)	0.071 (2)	0.0082 (17)	0.0045 (18)	−0.0242 (17)
O21	0.0370 (9)	0.0545 (11)	0.0527 (11)	−0.0122 (9)	−0.0152 (9)	0.0109 (9)
O22	0.0378 (9)	0.0463 (10)	0.0415 (10)	0.0118 (8)	−0.0044 (8)	0.0052 (9)
C23	0.0401 (13)	0.0290 (12)	0.0352 (13)	0.0008 (11)	−0.0051 (11)	0.0039 (11)
C24	0.0436 (14)	0.0420 (14)	0.0396 (14)	0.0014 (12)	−0.0144 (12)	0.0041 (12)
C25	0.0571 (16)	0.0446 (15)	0.0379 (14)	−0.0045 (14)	−0.0054 (13)	0.0147 (13)
O26	0.0440 (9)	0.0302 (8)	0.0436 (10)	0.0025 (8)	−0.0081 (8)	−0.0002 (8)
C27	0.0615 (17)	0.0313 (13)	0.0550 (17)	−0.0031 (13)	−0.0066 (15)	0.0010 (13)
O28	0.0450 (10)	0.0645 (12)	0.0449 (11)	−0.0010 (10)	0.0159 (9)	0.0023 (10)
O29	0.0655 (12)	0.0638 (12)	0.0371 (10)	−0.0013 (11)	−0.0084 (9)	−0.0092 (10)
O30	0.0416 (9)	0.0315 (9)	0.0652 (12)	−0.0020 (8)	0.0120 (9)	0.0047 (9)
C101	0.0273 (11)	0.0353 (13)	0.0396 (13)	0.0034 (11)	−0.0012 (10)	0.0088 (11)
C102	0.0275 (11)	0.0415 (14)	0.0369 (13)	0.0057 (11)	−0.0032 (11)	0.0077 (11)
C103	0.0279 (11)	0.0387 (14)	0.0347 (13)	0.0056 (11)	0.0016 (10)	0.0014 (11)
C104	0.0372 (13)	0.0483 (15)	0.0336 (13)	0.0042 (12)	0.0012 (11)	0.0003 (12)
C105	0.0530 (15)	0.0518 (16)	0.0294 (13)	0.0066 (14)	0.0030 (12)	−0.0071 (12)
C106	0.0601 (17)	0.0464 (15)	0.0360 (15)	0.0115 (14)	0.0040 (13)	−0.0036 (13)
C107	0.0330 (12)	0.0367 (14)	0.0484 (15)	0.0063 (11)	0.0059 (11)	0.0065 (12)
C108	0.0253 (11)	0.0383 (13)	0.0322 (12)	0.0021 (10)	0.0031 (10)	0.0046 (11)
C109	0.0241 (11)	0.0344 (13)	0.0414 (14)	0.0046 (10)	0.0037 (10)	0.0074 (11)
C110	0.0281 (11)	0.0352 (13)	0.0414 (14)	0.0000 (11)	−0.0030 (10)	0.0094 (11)
C111	0.0348 (12)	0.0458 (15)	0.0385 (14)	−0.0063 (12)	−0.0044 (11)	0.0080 (12)
C112	0.0294 (12)	0.0486 (15)	0.0372 (14)	−0.0045 (12)	0.0034 (11)	0.0051 (12)
C113	0.0245 (11)	0.0371 (13)	0.0394 (14)	−0.0051 (11)	−0.0016 (10)	−0.0044 (11)
C114	0.0230 (10)	0.0359 (13)	0.0362 (13)	0.0000 (10)	0.0012 (10)	0.0052 (11)
C115	0.0255 (11)	0.0390 (13)	0.0413 (14)	−0.0020 (10)	−0.0016 (10)	0.0124 (12)
O116	0.0297 (8)	0.0380 (9)	0.0477 (10)	−0.0018 (8)	−0.0077 (8)	0.0104 (8)
C117	0.0277 (11)	0.0394 (13)	0.0392 (13)	−0.0048 (11)	−0.0015 (10)	0.0068 (11)
C118	0.0395 (13)	0.0438 (15)	0.0430 (15)	−0.0031 (12)	−0.0103 (12)	0.0125 (12)
C119	0.0365 (13)	0.0523 (17)	0.0665 (18)	−0.0162 (13)	−0.0050 (13)	0.0141 (15)
C120	0.0595 (17)	0.0493 (16)	0.0526 (17)	−0.0082 (15)	−0.0133 (14)	0.0003 (15)
O121	0.0296 (8)	0.0420 (10)	0.0574 (11)	0.0039 (8)	0.0073 (8)	0.0030 (9)
O122	0.0346 (9)	0.0464 (10)	0.0451 (10)	−0.0110 (8)	−0.0007 (8)	0.0090 (9)
C123	0.0344 (13)	0.0455 (14)	0.0442 (15)	−0.0006 (12)	−0.0005 (12)	0.0139 (13)
C124	0.0373 (13)	0.0645 (18)	0.0535 (17)	0.0077 (14)	0.0114 (13)	0.0243 (15)
C125	0.0512 (15)	0.0552 (17)	0.0440 (16)	0.0020 (14)	−0.0010 (13)	0.0170 (14)
O126	0.0364 (9)	0.0465 (11)	0.0632 (12)	−0.0055 (9)	−0.0062 (9)	0.0198 (10)
C127	0.0651 (19)	0.0366 (15)	0.091 (2)	−0.0033 (15)	−0.0300 (18)	0.0114 (16)
O128	0.0539 (11)	0.0556 (12)	0.0606 (12)	0.0102 (10)	−0.0246 (10)	−0.0048 (10)
O129	0.0732 (13)	0.0809 (15)	0.0394 (11)	0.0235 (12)	0.0124 (10)	−0.0015 (11)
O130	0.0435 (10)	0.0446 (10)	0.0684 (13)	0.0108 (10)	−0.0083 (10)	0.0098 (10)
C201	0.0242 (10)	0.0241 (11)	0.0332 (12)	−0.0036 (9)	−0.0032 (9)	−0.0011 (10)
C202	0.0258 (11)	0.0342 (13)	0.0317 (12)	−0.0042 (10)	0.0007 (10)	0.0054 (10)
C203	0.0221 (10)	0.0327 (12)	0.0319 (12)	−0.0009 (10)	−0.0013 (9)	−0.0003 (10)
C204	0.0311 (12)	0.0380 (13)	0.0328 (13)	0.0021 (11)	0.0003 (10)	0.0021 (11)
C205	0.0386 (13)	0.0409 (14)	0.0347 (14)	0.0019 (12)	−0.0002 (11)	−0.0060 (12)
C206	0.0467 (14)	0.0327 (13)	0.0430 (15)	0.0012 (12)	−0.0009 (12)	−0.0077 (12)
C207	0.0309 (11)	0.0284 (12)	0.0456 (15)	−0.0010 (10)	−0.0011 (11)	−0.0011 (11)
C208	0.0244 (11)	0.0275 (12)	0.0338 (13)	−0.0012 (9)	0.0020 (9)	−0.0006 (10)
C209	0.0250 (10)	0.0260 (11)	0.0342 (12)	−0.0006 (10)	0.0006 (9)	−0.0002 (10)
C210	0.0343 (12)	0.0253 (11)	0.0397 (13)	0.0011 (10)	0.0067 (11)	0.0008 (10)
C211	0.0414 (13)	0.0350 (13)	0.0398 (14)	0.0091 (11)	0.0107 (11)	0.0098 (12)
C212	0.0357 (12)	0.0403 (14)	0.0334 (13)	0.0119 (12)	−0.0019 (11)	0.0038 (11)
C213	0.0290 (11)	0.0300 (12)	0.0306 (12)	0.0046 (10)	−0.0009 (10)	−0.0051 (10)
C214	0.0249 (10)	0.0250 (11)	0.0290 (12)	0.0020 (9)	−0.0023 (9)	0.0000 (10)
C215	0.0267 (11)	0.0292 (11)	0.0343 (13)	0.0038 (10)	0.0001 (10)	0.0052 (10)
O216	0.0308 (8)	0.0306 (8)	0.0412 (9)	0.0021 (7)	0.0081 (7)	0.0003 (8)
C217	0.0337 (12)	0.0356 (12)	0.0297 (12)	0.0064 (11)	−0.0020 (10)	0.0005 (11)
C218	0.0400 (13)	0.0301 (12)	0.0381 (14)	0.0033 (11)	0.0010 (11)	−0.0009 (11)
C219	0.0480 (15)	0.0493 (16)	0.0510 (16)	0.0171 (13)	0.0014 (13)	−0.0034 (14)
C220	0.0627 (17)	0.0482 (16)	0.0482 (17)	0.0133 (15)	−0.0033 (14)	−0.0098 (14)
O221	0.0321 (8)	0.0418 (10)	0.0555 (11)	−0.0053 (8)	−0.0143 (8)	0.0037 (9)
O222	0.0413 (9)	0.0389 (10)	0.0468 (10)	0.0157 (8)	0.0021 (8)	0.0068 (8)
C223	0.0354 (12)	0.0277 (11)	0.0305 (12)	0.0012 (10)	−0.0036 (10)	0.0003 (10)
C224	0.0330 (12)	0.0423 (14)	0.0335 (13)	0.0016 (11)	−0.0063 (10)	0.0022 (12)
C225	0.0475 (15)	0.0401 (14)	0.0448 (15)	0.0033 (13)	−0.0011 (12)	0.0123 (13)
O226	0.0368 (9)	0.0287 (8)	0.0430 (10)	0.0048 (8)	−0.0024 (8)	−0.0024 (7)
C227	0.0568 (16)	0.0286 (12)	0.0566 (17)	−0.0002 (13)	−0.0030 (14)	−0.0064 (12)
O228	0.0428 (10)	0.0464 (10)	0.0579 (12)	−0.0045 (9)	0.0213 (9)	0.0010 (9)
O229	0.0563 (11)	0.0636 (12)	0.0394 (10)	−0.0075 (11)	−0.0102 (9)	−0.0074 (10)
O230	0.0436 (10)	0.0302 (9)	0.0669 (13)	−0.0077 (8)	0.0111 (10)	−0.0002 (9)

### Geometric parameters (Å, °)

**Table:**

C1—C2	1.502 (3)	C112—H1121	0.978
C1—C14	1.543 (3)	C113—C114	1.541 (3)
C1—C23	1.563 (3)	C113—O121	1.215 (3)
C1—H11	0.994	C114—C115	1.567 (3)
C2—C3	1.337 (3)	C115—O116	1.468 (3)
C2—H21	0.968	C115—C117	1.494 (3)
C3—C4	1.495 (3)	C115—H1151	0.979
C3—C8	1.511 (3)	C117—C118	1.329 (3)
C4—C5	1.496 (4)	C117—H1171	0.935
C4—O28	1.216 (3)	C118—C119	1.503 (3)
C5—C6	1.452 (4)	C118—C120	1.505 (4)
C5—O29	1.451 (3)	C119—H1191	0.959
C5—H51	1.002	C119—H1192	0.930
C6—C7	1.506 (3)	C119—H1193	0.954
C6—O29	1.441 (3)	C120—H1201	0.961
C6—H61	0.975	C120—H1202	0.979
C7—C8	1.532 (3)	C120—H1203	0.946
C7—O30	1.422 (3)	C123—C124	1.525 (3)
C7—H71	0.986	C123—C125	1.534 (3)
C8—C9	1.537 (3)	C123—O126	1.441 (3)
C8—H81	0.989	C124—H1241	0.979
C9—C10	1.526 (3)	C124—H1242	0.985
C9—C14	1.550 (3)	C124—H1243	0.976
C9—H91	0.984	C125—H1251	0.990
C10—C11	1.509 (3)	C125—H1252	0.998
C10—O16	1.440 (3)	C125—H1253	0.954
C10—H101	0.995	O126—C127	1.417 (3)
C11—C12	1.458 (3)	C127—H1271	0.968
C11—O22	1.444 (3)	C127—H1272	0.993
C11—H111	0.985	C127—H1273	0.950
C12—C13	1.486 (3)	O130—H1301	0.81 (3)
C12—O22	1.450 (3)	C201—C202	1.516 (3)
C12—H121	0.983	C201—C214	1.548 (3)
C13—C14	1.536 (3)	C201—C223	1.561 (3)
C13—O21	1.218 (3)	C201—H2011	0.977
C14—C15	1.574 (3)	C202—C203	1.334 (3)
C15—O16	1.459 (3)	C202—H2021	0.926
C15—C17	1.489 (3)	C203—C204	1.494 (3)
C15—H151	1.019	C203—C208	1.516 (3)
C17—C18	1.337 (3)	C204—C205	1.490 (3)
C17—H171	1.003	C204—O228	1.216 (3)
C18—C19	1.496 (3)	C205—C206	1.464 (3)
C18—C20	1.503 (4)	C205—O229	1.453 (3)
C19—H191	0.951	C205—H2051	1.035
C19—H192	0.948	C206—C207	1.516 (3)
C19—H193	0.962	C206—O229	1.434 (3)
C20—H201	0.976	C206—H2061	0.958
C20—H202	0.974	C207—C208	1.528 (3)
C20—H203	0.961	C207—O230	1.429 (3)
C23—C24	1.518 (3)	C207—H2071	0.962
C23—C25	1.529 (3)	C208—C209	1.525 (3)
C23—O26	1.437 (3)	C208—H2081	0.993
C24—H241	0.951	C209—C210	1.536 (3)
C24—H242	0.984	C209—C214	1.557 (3)
C24—H243	0.968	C209—H2091	0.973
C25—H251	0.977	C210—C211	1.500 (3)
C25—H252	0.976	C210—O216	1.449 (3)
C25—H253	0.992	C210—H2101	0.974
O26—C27	1.425 (3)	C211—C212	1.464 (3)
C27—H271	0.964	C211—O222	1.444 (3)
C27—H272	0.974	C211—H2111	0.962
C27—H273	0.977	C212—C213	1.501 (3)
O30—H301	0.91 (3)	C212—O222	1.447 (3)
C101—C102	1.501 (3)	C212—H2121	0.978
C101—C114	1.548 (3)	C213—C214	1.542 (3)
C101—C123	1.559 (3)	C213—O221	1.209 (3)
C101—H1011	0.979	C214—C215	1.575 (3)
C102—C103	1.335 (3)	C215—O216	1.461 (3)
C102—H1021	0.925	C215—C217	1.499 (3)
C103—C104	1.496 (3)	C215—H2151	0.981
C103—C108	1.519 (3)	C217—C218	1.330 (3)
C104—C105	1.495 (4)	C217—H2171	0.934
C104—O128	1.212 (3)	C218—C219	1.494 (3)
C105—C106	1.464 (4)	C218—C220	1.504 (3)
C105—O129	1.452 (3)	C219—H2191	0.982
C105—H1051	1.025	C219—H2192	0.927
C106—C107	1.517 (4)	C219—H2193	0.956
C106—O129	1.431 (3)	C220—H2201	0.957
C106—H1061	1.006	C220—H2202	0.948
C107—C108	1.531 (3)	C220—H2203	1.004
C107—O130	1.421 (3)	C223—C224	1.523 (3)
C107—H1071	0.979	C223—C225	1.525 (3)
C108—C109	1.526 (3)	C223—O226	1.440 (3)
C108—H1081	1.006	C224—H2241	0.984
C109—C110	1.525 (3)	C224—H2242	0.959
C109—C114	1.545 (3)	C224—H2243	0.941
C109—H1091	0.959	C225—H2251	0.963
C110—C111	1.518 (3)	C225—H2252	0.968
C110—O116	1.449 (3)	C225—H2253	0.966
C110—H1101	0.960	O226—C227	1.420 (3)
C111—C112	1.461 (3)	C227—H2271	0.976
C111—O122	1.441 (3)	C227—H2272	0.953
C111—H1111	0.980	C227—H2273	0.970
C112—C113	1.511 (3)	O230—H2301	0.76 (3)
C112—O122	1.437 (3)		
			
O16···C104	2.941 (3)	O128···C227	3.489 (3)
O16···C105	3.115 (3)	O129···C11^vii^	3.128 (3)
O16···C103	3.230 (3)	O129···C12^vii^	3.224 (3)
O16···C108	3.293 (3)	O129···C227^vii^	3.476 (3)
O16···O122	3.298 (2)	O130···C19	3.489 (3)
O16···O128	3.415 (2)	O216···C204^viii^	3.003 (3)
O21···O130^i^	2.796 (2)	O216···C205^viii^	3.099 (3)
O21···C220^ii^	3.568 (3)	O216···C208^viii^	3.319 (2)
O22···C124^i^	3.429 (3)	O216···C203^viii^	3.350 (2)
O22···O129^ii^	3.519 (3)	O216···O228^viii^	3.459 (2)
O28···O116^i^	3.251 (2)	O221···O230^ix^	2.809 (2)
O28···C111^i^	3.298 (3)	O221···C20^ii^	3.267 (4)
O28···O228^iii^	3.495 (2)	O221···C207^ix^	3.582 (3)
O28···C110^i^	3.504 (3)	O222···C224^ix^	3.526 (3)
O28···C212^iv^	3.570 (3)	O228···C127^i^	3.409 (3)
O30···O121	2.828 (2)	O228···C211^ix^	3.455 (3)
O30···C225	3.426 (3)	O229···C24^v^	3.555 (3)
O30···C27^v^	3.584 (3)	O230···C20^x^	3.542 (4)
O116···C4^vi^	2.851 (3)	C3···C110^i^	3.595 (3)
O116···C5^vi^	3.052 (3)	C4···C110^i^	3.448 (3)
O116···C3^vi^	3.228 (3)	C6···C27^v^	3.538 (4)
O116···C8^vi^	3.318 (2)	C10···C104	3.586 (3)
O121···C25^v^	3.254 (3)	C25···C224^xi^	3.574 (4)
O122···C9	3.547 (3)	C106···C227^vii^	3.534 (4)
O122···C10	3.560 (3)	C120···C220^xii^	3.492 (4)
O128···O129^ii^	3.216 (3)	C204···C210^ix^	3.557 (3)
O128···C11	3.400 (3)		
			
C2—C1—C14	109.40 (18)	C112—C113—C114	118.72 (19)
C2—C1—C23	110.25 (18)	C112—C113—O121	118.7 (2)
C14—C1—C23	120.75 (18)	C114—C113—O121	122.5 (2)
C2—C1—H11	104.4	C113—C114—C109	108.99 (18)
C14—C1—H11	106.6	C113—C114—C101	108.40 (17)
C23—C1—H11	104.1	C109—C114—C101	113.7 (2)
C1—C2—C3	127.2 (2)	C113—C114—C115	107.46 (18)
C1—C2—H21	116.8	C109—C114—C115	99.08 (17)
C3—C2—H21	115.9	C101—C114—C115	118.65 (18)
C2—C3—C4	117.8 (2)	C114—C115—O116	104.66 (17)
C2—C3—C8	124.5 (2)	C114—C115—C117	117.19 (19)
C4—C3—C8	117.6 (2)	O116—C115—C117	110.96 (19)
C3—C4—C5	116.6 (2)	C114—C115—H1151	105.9
C3—C4—O28	123.1 (2)	O116—C115—H1151	106.6
C5—C4—O28	120.2 (2)	C117—C115—H1151	110.8
C4—C5—C6	119.9 (2)	C115—O116—C110	109.47 (15)
C4—C5—O29	110.4 (2)	C115—C117—C118	126.1 (2)
C6—C5—O29	59.53 (16)	C115—C117—H1171	116.8
C4—C5—H51	115.9	C118—C117—H1171	117.1
C6—C5—H51	122.7	C117—C118—C119	124.4 (3)
O29—C5—H51	111.3	C117—C118—C120	121.9 (2)
C5—C6—C7	122.6 (2)	C119—C118—C120	113.7 (2)
C5—C6—O29	60.23 (16)	C118—C119—H1191	110.4
C7—C6—O29	116.3 (2)	C118—C119—H1192	110.4
C5—C6—H61	117.9	H1191—C119—H1192	107.2
C7—C6—H61	114.0	C118—C119—H1193	112.1
O29—C6—H61	114.5	H1191—C119—H1193	108.1
C6—C7—C8	112.45 (19)	H1192—C119—H1193	108.3
C6—C7—O30	108.22 (19)	C118—C120—H1201	113.5
C8—C7—O30	108.25 (19)	C118—C120—H1202	112.3
C6—C7—H71	110.2	H1201—C120—H1202	109.0
C8—C7—H71	109.4	C118—C120—H1203	111.2
O30—C7—H71	108.2	H1201—C120—H1203	104.0
C7—C8—C3	112.96 (19)	H1202—C120—H1203	106.3
C7—C8—C9	110.47 (17)	C111—O122—C112	61.03 (15)
C3—C8—C9	109.87 (18)	C101—C123—C124	114.9 (2)
C7—C8—H81	106.3	C101—C123—C125	108.69 (19)
C3—C8—H81	108.3	C124—C123—C125	108.8 (2)
C9—C8—H81	108.8	C101—C123—O126	110.0 (2)
C8—C9—C10	121.32 (18)	C124—C123—O126	104.5 (2)
C8—C9—C14	116.39 (17)	C125—C123—O126	109.9 (2)
C10—C9—C14	100.32 (17)	C123—C124—H1241	110.7
C8—C9—H91	106.4	C123—C124—H1242	107.9
C10—C9—H91	103.8	H1241—C124—H1242	109.4
C14—C9—H91	107.3	C123—C124—H1243	110.0
C9—C10—C11	114.65 (18)	H1241—C124—H1243	111.3
C9—C10—O16	103.11 (17)	H1242—C124—H1243	107.4
C11—C10—O16	104.41 (18)	C123—C125—H1251	107.3
C9—C10—H101	111.9	C123—C125—H1252	111.0
C11—C10—H101	110.9	H1251—C125—H1252	108.6
O16—C10—H101	111.4	C123—C125—H1253	108.3
C10—C11—C12	116.1 (2)	H1251—C125—H1253	110.9
C10—C11—O22	118.42 (19)	H1252—C125—H1253	110.7
C12—C11—O22	59.93 (14)	C123—O126—C127	116.83 (19)
C10—C11—H111	115.2	O126—C127—H1271	107.4
C12—C11—H111	119.2	O126—C127—H1272	108.5
O22—C11—H111	117.0	H1271—C127—H1272	105.2
C11—C12—C13	118.1 (2)	O126—C127—H1273	111.2
C11—C12—O22	59.54 (15)	H1271—C127—H1273	110.3
C13—C12—O22	114.42 (19)	H1272—C127—H1273	114.0
C11—C12—H121	120.1	C105—O129—C106	61.00 (16)
C13—C12—H121	116.8	C107—O130—H1301	108 (2)
O22—C12—H121	114.7	C202—C201—C214	108.56 (17)
C12—C13—C14	119.39 (19)	C202—C201—C223	110.84 (18)
C12—C13—O21	118.7 (2)	C214—C201—C223	120.23 (17)
C14—C13—O21	121.7 (2)	C202—C201—H2011	105.0
C1—C14—C13	108.69 (17)	C214—C201—H2011	108.2
C1—C14—C9	113.41 (18)	C223—C201—H2011	102.8
C13—C14—C9	111.30 (18)	C201—C202—C203	127.3 (2)
C1—C14—C15	119.69 (18)	C201—C202—H2021	116.7
C13—C14—C15	106.28 (17)	C203—C202—H2021	116.0
C9—C14—C15	96.88 (16)	C202—C203—C204	118.0 (2)
C14—C15—O16	104.86 (16)	C202—C203—C208	124.4 (2)
C14—C15—C17	119.49 (18)	C204—C203—C208	117.48 (19)
O16—C15—C17	108.69 (18)	C203—C204—C205	117.5 (2)
C14—C15—H151	107.9	C203—C204—O228	123.3 (2)
O16—C15—H151	106.6	C205—C204—O228	119.1 (2)
C17—C15—H151	108.6	C204—C205—C206	119.4 (2)
C15—O16—C10	109.47 (15)	C204—C205—O229	110.7 (2)
C15—C17—C18	124.1 (2)	C206—C205—O229	58.91 (15)
C15—C17—H171	116.8	C204—C205—H2051	117.8
C18—C17—H171	119.0	C206—C205—H2051	121.0
C17—C18—C19	124.7 (2)	O229—C205—H2051	111.4
C17—C18—C20	121.3 (2)	C205—C206—C207	122.1 (2)
C19—C18—C20	114.0 (2)	C205—C206—O229	60.15 (15)
C18—C19—H191	109.9	C207—C206—O229	116.5 (2)
C18—C19—H192	109.1	C205—C206—H2061	117.8
H191—C19—H192	104.7	C207—C206—H2061	113.5
C18—C19—H193	112.2	O229—C206—H2061	116.5
H191—C19—H193	111.5	C206—C207—C208	113.16 (19)
H192—C19—H193	109.2	C206—C207—O230	108.34 (18)
C18—C20—H201	108.6	C208—C207—O230	107.21 (19)
C18—C20—H202	109.5	C206—C207—H2071	107.5
H201—C20—H202	109.6	C208—C207—H2071	108.9
C18—C20—H203	110.6	O230—C207—H2071	111.7
H201—C20—H203	107.8	C207—C208—C203	112.68 (19)
H202—C20—H203	110.7	C207—C208—C209	110.88 (17)
C12—O22—C11	60.52 (15)	C203—C208—C209	109.91 (17)
C1—C23—C24	114.37 (18)	C207—C208—H2081	104.7
C1—C23—C25	108.61 (19)	C203—C208—H2081	108.1
C24—C23—C25	108.9 (2)	C209—C208—H2081	110.4
C1—C23—O26	109.85 (18)	C208—C209—C210	121.85 (18)
C24—C23—O26	105.44 (18)	C208—C209—C214	115.92 (17)
C25—C23—O26	109.54 (19)	C210—C209—C214	100.26 (17)
C23—C24—H241	108.0	C208—C209—H2091	106.2
C23—C24—H242	108.9	C210—C209—H2091	104.8
H241—C24—H242	110.9	C214—C209—H2091	106.5
C23—C24—H243	111.2	C209—C210—C211	115.33 (19)
H241—C24—H243	108.9	C209—C210—O216	102.06 (16)
H242—C24—H243	108.9	C211—C210—O216	104.85 (19)
C23—C25—H251	111.3	C209—C210—H2101	113.1
C23—C25—H252	108.7	C211—C210—H2101	110.9
H251—C25—H252	111.2	O216—C210—H2101	109.8
C23—C25—H253	111.3	C210—C211—C212	117.3 (2)
H251—C25—H253	107.1	C210—C211—O222	118.8 (2)
H252—C25—H253	107.1	C212—C211—O222	59.70 (15)
C23—O26—C27	117.18 (19)	C210—C211—H2111	117.9
O26—C27—H271	111.1	C212—C211—H2111	116.9
O26—C27—H272	114.2	O222—C211—H2111	113.2
H271—C27—H272	107.5	C211—C212—C213	117.8 (2)
O26—C27—H273	110.4	C211—C212—O222	59.48 (15)
H271—C27—H273	105.9	C213—C212—O222	114.29 (19)
H272—C27—H273	107.3	C211—C212—H2121	119.0
C5—O29—C6	60.24 (16)	C213—C212—H2121	117.2
C7—O30—H301	111.3 (20)	O222—C212—H2121	116.0
C102—C101—C114	107.85 (18)	C212—C213—C214	117.80 (19)
C102—C101—C123	110.4 (2)	C212—C213—O221	119.4 (2)
C114—C101—C123	120.67 (18)	C214—C213—O221	122.7 (2)
C102—C101—H1011	106.7	C213—C214—C201	108.10 (17)
C114—C101—H1011	105.2	C213—C214—C209	108.52 (17)
C123—C101—H1011	105.1	C201—C214—C209	113.13 (17)
C101—C102—C103	126.6 (2)	C213—C214—C215	107.67 (17)
C101—C102—H1021	116.8	C201—C214—C215	119.95 (17)
C103—C102—H1021	116.7	C209—C214—C215	98.77 (16)
C102—C103—C104	118.1 (2)	C214—C215—O216	106.02 (16)
C102—C103—C108	124.7 (2)	C214—C215—C217	118.49 (18)
C104—C103—C108	117.2 (2)	O216—C215—C217	108.48 (18)
C103—C104—C105	117.1 (2)	C214—C215—H2151	107.6
C103—C104—O128	123.4 (2)	O216—C215—H2151	106.5
C105—C104—O128	119.4 (2)	C217—C215—H2151	109.1
C104—C105—C106	119.3 (2)	C215—O216—C210	109.04 (15)
C104—C105—O129	109.7 (2)	C215—C217—C218	125.8 (2)
C106—C105—O129	58.80 (16)	C215—C217—H2171	116.2
C104—C105—H1051	119.6	C218—C217—H2171	117.8
C106—C105—H1051	120.9	C217—C218—C219	125.5 (2)
O129—C105—H1051	96.3	C217—C218—C220	120.9 (2)
C105—C106—C107	121.4 (2)	C219—C218—C220	113.6 (2)
C105—C106—O129	60.20 (16)	C218—C219—H2191	110.6
C107—C106—O129	116.6 (2)	C218—C219—H2192	110.3
C105—C106—H1061	117.3	H2191—C219—H2192	109.1
C107—C106—H1061	115.9	C218—C219—H2193	108.4
O129—C106—H1061	113.2	H2191—C219—H2193	109.8
C106—C107—C108	111.81 (19)	H2192—C219—H2193	108.5
C106—C107—O130	108.5 (2)	C218—C220—H2201	110.0
C108—C107—O130	107.4 (2)	C218—C220—H2202	107.3
C106—C107—H1071	106.7	H2201—C220—H2202	109.3
C108—C107—H1071	110.1	C218—C220—H2203	110.8
O130—C107—H1071	112.4	H2201—C220—H2203	110.4
C107—C108—C103	111.20 (19)	H2202—C220—H2203	109.1
C107—C108—C109	111.98 (18)	C212—O222—C211	60.82 (14)
C103—C108—C109	110.86 (19)	C201—C223—C224	113.69 (18)
C107—C108—H1081	108.2	C201—C223—C225	109.92 (18)
C103—C108—H1081	105.8	C224—C223—C225	108.77 (19)
C109—C108—H1081	108.5	C201—C223—O226	109.98 (17)
C108—C109—C110	122.16 (19)	C224—C223—O226	104.24 (18)
C108—C109—C114	115.27 (18)	C225—C223—O226	110.09 (18)
C110—C109—C114	100.41 (19)	C223—C224—H2241	109.7
C108—C109—H1091	107.1	C223—C224—H2242	110.7
C110—C109—H1091	105.8	H2241—C224—H2242	106.7
C114—C109—H1091	104.6	C223—C224—H2243	110.1
C109—C110—C111	114.85 (18)	H2241—C224—H2243	108.1
C109—C110—O116	103.56 (18)	H2242—C224—H2243	111.4
C111—C110—O116	103.92 (19)	C223—C225—H2251	107.8
C109—C110—H1101	114.2	C223—C225—H2252	110.4
C111—C110—H1101	110.9	H2251—C225—H2252	107.6
O116—C110—H1101	108.4	C223—C225—H2253	114.6
C110—C111—C112	115.5 (2)	H2251—C225—H2253	107.0
C110—C111—O122	118.6 (2)	H2252—C225—H2253	109.3
C112—C111—O122	59.32 (15)	C223—O226—C227	117.00 (17)
C110—C111—H1111	116.6	O226—C227—H2271	108.8
C112—C111—H1111	119.6	O226—C227—H2272	111.3
O122—C111—H1111	115.0	H2271—C227—H2272	109.2
C111—C112—C113	118.1 (2)	O226—C227—H2273	111.4
C111—C112—O122	59.64 (15)	H2271—C227—H2273	108.1
C113—C112—O122	114.1 (2)	H2272—C227—H2273	108.0
C111—C112—H1121	121.2	C205—O229—C206	60.93 (15)
C113—C112—H1121	116.3	C207—O230—H2301	112 (3)
O122—C112—H1121	114.0		
			
O16—C10—C9—C8	−174.4 (2)	C10—C11—C12—C13	5.9 (3)
O16—C10—C9—C14	−44.6 (2)	C11—O22—C12—C13	109.4 (2)
O16—C10—C11—O22	138.8 (2)	C11—C10—O16—C15	−98.6 (2)
O16—C10—C11—C12	70.5 (2)	C11—C10—C9—C14	68.3 (2)
O16—C15—C14—C1	−158.0 (2)	C11—C12—C13—C14	−3.5 (3)
O16—C15—C14—C9	−36.0 (2)	C12—C13—C14—C15	−69.6 (2)
O16—C15—C14—C13	78.6 (2)	C13—C14—C1—C23	145.4 (2)
O16—C15—C17—C18	100.8 (3)	C13—C14—C15—C17	−43.5 (3)
O21—C13—C12—O22	114.4 (2)	C14—C1—C23—C24	60.0 (3)
O21—C13—C12—C11	−178.5 (2)	C14—C1—C23—C25	−178.1 (2)
O21—C13—C14—C1	−24.9 (3)	C14—C15—C17—C18	−139.1 (2)
O21—C13—C14—C9	−150.5 (2)	C15—C14—C1—C23	23.2 (3)
O21—C13—C14—C15	105.2 (2)	C15—C17—C18—C19	0.7 (4)
O22—C11—C10—C9	26.8 (3)	C15—C17—C18—C20	179.0 (2)
O22—C11—C12—C13	−103.3 (2)	C24—C23—O26—C27	170.2 (2)
O22—C12—C11—C10	109.2 (2)	C25—C23—O26—C27	53.2 (3)
O22—C12—C13—C14	−70.6 (2)	C101—C102—C103—C104	−179.7 (2)
O26—C23—C1—C2	172.6 (2)	C101—C102—C103—C108	2.3 (4)
O26—C23—C1—C14	−58.3 (3)	C101—C114—C109—C108	−54.2 (2)
O28—C4—C3—C2	−16.9 (3)	C101—C114—C109—C110	172.5 (2)
O28—C4—C3—C8	159.9 (2)	C101—C114—C113—C112	164.9 (2)
O28—C4—C5—O29	106.8 (2)	C101—C114—C115—C117	80.5 (3)
O28—C4—C5—C6	172.6 (2)	C101—C123—O126—C127	−69.5 (3)
O29—C5—C4—C3	−71.1 (3)	C102—C101—C114—C109	45.0 (2)
O29—C5—C6—C7	103.9 (2)	C102—C101—C114—C113	−76.4 (2)
O29—C6—C5—C4	−97.4 (2)	C102—C101—C114—C115	160.8 (2)
O29—C6—C7—O30	−151.7 (2)	C102—C101—C123—C124	−71.8 (3)
O29—C6—C7—C8	88.8 (2)	C102—C101—C123—C125	50.3 (3)
O30—C7—C6—C5	138.3 (2)	C102—C103—C104—C105	163.8 (2)
O30—C7—C8—C3	−163.8 (2)	C102—C103—C108—C107	−132.4 (2)
O30—C7—C8—C9	72.7 (2)	C102—C103—C108—C109	−7.2 (3)
O116—C110—C109—C108	−172.3 (2)	C103—C102—C101—C114	−21.2 (3)
O116—C110—C109—C114	−43.4 (2)	C103—C102—C101—C123	112.6 (3)
O116—C110—C111—O122	139.9 (2)	C103—C104—C105—C106	−11.4 (3)
O116—C110—C111—C112	72.5 (2)	C103—C108—C107—C106	−50.5 (3)
O116—C115—C114—C101	−156.1 (2)	C103—C108—C109—C110	154.5 (2)
O116—C115—C114—C109	−32.7 (2)	C103—C108—C109—C114	32.3 (3)
O116—C115—C114—C113	80.6 (2)	C104—C103—C108—C107	49.6 (3)
O116—C115—C117—C118	102.7 (3)	C104—C103—C108—C109	174.8 (2)
O121—C113—C112—O122	107.4 (2)	C104—C105—O129—C106	113.0 (2)
O121—C113—C112—C111	174.5 (2)	C104—C105—C106—C107	8.3 (3)
O121—C113—C114—C101	−18.0 (3)	C105—O129—C106—C107	−112.6 (2)
O121—C113—C114—C109	−142.2 (2)	C105—C104—C103—C108	−18.1 (3)
O121—C113—C114—C115	111.3 (2)	C105—C106—C107—C108	23.3 (3)
O122—C111—C110—C109	27.5 (3)	C106—C107—C108—C109	−175.1 (2)
O122—C111—C112—C113	−102.9 (2)	C107—C108—C109—C110	−80.7 (3)
O122—C112—C111—C110	109.5 (2)	C107—C108—C109—C114	157.1 (2)
O122—C112—C113—C114	−75.4 (3)	C108—C109—C110—C111	−59.7 (3)
O126—C123—C101—C102	170.7 (2)	C108—C109—C114—C113	66.8 (2)
O126—C123—C101—C114	−62.4 (3)	C108—C109—C114—C115	178.9 (2)
O128—C104—C103—C102	−13.4 (4)	C109—C110—O116—C115	23.0 (2)
O128—C104—C103—C108	164.7 (2)	C109—C110—C111—C112	−39.9 (3)
O128—C104—C105—O129	101.4 (3)	C109—C114—C101—C123	−83.1 (3)
O128—C104—C105—C106	165.9 (2)	C109—C114—C113—C112	40.7 (3)
O129—C105—C104—C103	−75.9 (3)	C109—C114—C115—C117	−156.1 (2)
O129—C105—C106—C107	104.8 (3)	C110—O116—C115—C114	6.6 (2)
O129—C106—C105—C104	−96.4 (2)	C110—O116—C115—C117	133.9 (2)
O129—C106—C107—O130	−148.7 (2)	C110—C109—C114—C113	−66.4 (2)
O129—C106—C107—C108	93.1 (2)	C110—C109—C114—C115	45.7 (2)
O130—C107—C106—C105	141.5 (2)	C110—C111—O122—C112	−104.3 (2)
O130—C107—C108—C103	−169.4 (2)	C110—C111—C112—C113	6.6 (3)
O130—C107—C108—C109	66.0 (2)	C111—O122—C112—C113	109.6 (2)
O216—C210—C209—C208	−176.8 (2)	C111—C110—O116—C115	−97.4 (2)
O216—C210—C209—C214	−47.3 (2)	C111—C110—C109—C114	69.2 (2)
O216—C210—C211—O222	144.3 (2)	C111—C112—C113—C114	−8.3 (3)
O216—C210—C211—C212	75.7 (2)	C112—C113—C114—C115	−65.7 (3)
O216—C215—C214—C201	−149.8 (2)	C113—C114—C101—C123	155.6 (2)
O216—C215—C214—C209	−26.6 (2)	C113—C114—C115—C117	−42.7 (3)
O216—C215—C214—C213	86.1 (2)	C114—C101—C123—C124	55.1 (3)
O216—C215—C217—C218	99.9 (3)	C114—C101—C123—C125	177.2 (2)
O221—C213—C212—O222	104.0 (2)	C114—C115—C117—C118	−137.2 (2)
O221—C213—C212—C211	171.0 (2)	C115—C114—C101—C123	32.8 (3)
O221—C213—C214—C201	−14.3 (3)	C115—C117—C118—C119	−3.8 (4)
O221—C213—C214—C209	−137.4 (2)	C115—C117—C118—C120	173.9 (2)
O221—C213—C214—C215	116.6 (2)	C124—C123—O126—C127	166.7 (2)
O222—C211—C210—C209	32.9 (3)	C125—C123—O126—C127	50.1 (3)
O222—C211—C212—C213	−103.2 (2)	C201—C202—C203—C204	178.7 (2)
O222—C212—C211—C210	109.1 (2)	C201—C202—C203—C208	2.6 (3)
O222—C212—C213—C214	−78.9 (2)	C201—C214—C209—C208	−54.8 (2)
O226—C223—C201—C202	172.2 (2)	C201—C214—C209—C210	172.0 (2)
O226—C223—C201—C214	−59.8 (2)	C201—C214—C213—C212	168.7 (2)
O228—C204—C203—C202	−17.2 (3)	C201—C214—C215—C217	88.1 (2)
O228—C204—C203—C208	159.2 (2)	C201—C223—O226—C227	−67.7 (2)
O228—C204—C205—O229	108.3 (2)	C202—C201—C214—C209	40.4 (2)
O228—C204—C205—C206	173.4 (2)	C202—C201—C214—C213	−79.8 (2)
O229—C205—C204—C203	−70.0 (3)	C202—C201—C214—C215	156.3 (2)
O229—C205—C206—C207	104.4 (2)	C202—C201—C223—C224	−71.4 (2)
O229—C206—C205—C204	−97.7 (2)	C202—C201—C223—C225	50.8 (2)
O229—C206—C207—O230	−152.9 (2)	C202—C203—C204—C205	161.0 (2)
O229—C206—C207—C208	88.3 (2)	C202—C203—C208—C207	−136.4 (2)
O230—C207—C206—C205	137.2 (2)	C202—C203—C208—C209	−12.2 (3)
O230—C207—C208—C203	−163.3 (2)	C203—C202—C201—C214	−16.9 (3)
O230—C207—C208—C209	73.1 (2)	C203—C202—C201—C223	117.2 (2)
C1—C2—C3—C4	179.7 (2)	C203—C204—C205—C206	−4.9 (3)
C1—C2—C3—C8	3.2 (4)	C203—C208—C207—C206	−43.8 (3)
C1—C14—C9—C8	−52.5 (2)	C203—C208—C209—C210	159.9 (2)
C1—C14—C9—C10	174.6 (2)	C203—C208—C209—C214	37.5 (2)
C1—C14—C13—C12	160.3 (2)	C204—C203—C208—C207	47.5 (2)
C1—C14—C15—C17	79.9 (3)	C204—C203—C208—C209	171.7 (2)
C1—C23—O26—C27	−66.1 (2)	C204—C205—O229—C206	112.7 (2)
C2—C1—C14—C9	39.3 (2)	C204—C205—C206—C207	6.6 (3)
C2—C1—C14—C13	−85.0 (2)	C205—O229—C206—C207	−113.4 (2)
C2—C1—C14—C15	152.7 (2)	C205—C204—C203—C208	−22.6 (3)
C2—C1—C23—C24	−69.2 (2)	C205—C206—C207—C208	18.4 (3)
C2—C1—C23—C25	52.7 (2)	C206—C207—C208—C209	−167.5 (2)
C2—C3—C4—C5	161.0 (2)	C207—C208—C209—C210	−74.9 (2)
C2—C3—C8—C7	−135.7 (2)	C207—C208—C209—C214	162.8 (2)
C2—C3—C8—C9	−11.9 (3)	C208—C209—C210—C211	−63.7 (3)
C3—C2—C1—C14	−17.0 (3)	C208—C209—C214—C213	65.2 (2)
C3—C2—C1—C23	118.1 (2)	C208—C209—C214—C215	177.2 (2)
C3—C4—C5—C6	−5.3 (3)	C209—C210—O216—C215	31.0 (2)
C3—C8—C7—C6	−44.4 (3)	C209—C210—C211—C212	−35.7 (3)
C3—C8—C9—C10	158.4 (2)	C209—C214—C201—C223	−88.7 (2)
C3—C8—C9—C14	36.0 (2)	C209—C214—C213—C212	45.7 (3)
C4—C3—C8—C7	47.8 (3)	C209—C214—C215—C217	−148.7 (2)
C4—C3—C8—C9	171.6 (2)	C210—O216—C215—C214	−2.3 (2)
C4—C5—O29—C6	113.5 (2)	C210—O216—C215—C217	125.9 (2)
C4—C5—C6—C7	6.5 (4)	C210—C209—C214—C213	−68.0 (2)
C5—O29—C6—C7	−114.2 (2)	C210—C209—C214—C215	44.1 (2)
C5—C4—C3—C8	−22.2 (3)	C210—C211—O222—C212	−106.5 (2)
C5—C6—C7—C8	18.8 (3)	C210—C211—C212—C213	5.8 (3)
C6—C7—C8—C9	−167.9 (2)	C211—O222—C212—C213	109.2 (2)
C7—C8—C9—C10	−76.3 (2)	C211—C210—O216—C215	−89.6 (2)
C7—C8—C9—C14	161.3 (2)	C211—C210—C209—C214	65.7 (2)
C8—C9—C10—C11	−61.5 (3)	C211—C212—C213—C214	−12.0 (3)
C8—C9—C14—C13	70.4 (2)	C212—C213—C214—C215	−60.3 (2)
C8—C9—C14—C15	−179.1 (2)	C213—C214—C201—C223	151.1 (2)
C9—C10—O16—C15	21.5 (2)	C213—C214—C215—C217	−35.9 (2)
C9—C10—C11—C12	−41.6 (3)	C214—C201—C223—C224	56.6 (3)
C9—C14—C1—C23	−90.2 (2)	C214—C201—C223—C225	178.8 (2)
C9—C14—C13—C12	34.7 (3)	C214—C215—C217—C218	−139.3 (2)
C9—C14—C15—C17	−158.1 (2)	C215—C214—C201—C223	27.3 (3)
C10—O16—C15—C14	9.9 (2)	C215—C217—C218—C219	3.5 (4)
C10—O16—C15—C17	138.7 (2)	C215—C217—C218—C220	−175.9 (2)
C10—C9—C14—C13	−62.5 (2)	C224—C223—O226—C227	170.1 (2)
C10—C9—C14—C15	48.0 (2)	C225—C223—O226—C227	53.6 (2)
C10—C11—O22—C12	−105.4 (2)		

Symmetry codes: (i) *x*−1, *y*, *z*; (ii) *x*−1/2, −*y*+1/2, −*z*+1; (iii) −*x*, *y*−1/2, −*z*+3/2; (iv) −*x*+1/2, −*y*+1, *z*+1/2; (v) −*x*+1, *y*+1/2, −*z*+3/2; (vi) *x*+1, *y*, *z*; (vii) *x*+1/2, −*y*+1/2, −*z*+1; (viii) *x*+1/2, −*y*+3/2, −*z*+1; (ix) *x*−1/2, −*y*+3/2, −*z*+1; (x) *x*, *y*+1, *z*; (xi) −*x*+1, *y*−1/2, −*z*+3/2; (xii) −*x*+3/2, −*y*+1, *z*+1/2.

### Hydrogen-bond geometry (Å, °)

**Table:**

*D*—H···*A*	*D*—H	H···*A*	*D*···*A*	*D*—H···*A*
O30—H301···O121	0.91 (3)	1.92 (3)	2.828 (3)	172 (3)
O130—H1301···O21^vi^	0.81 (3)	2.04 (3)	2.796 (3)	155 (3)
O230—H2301···O221^viii^	0.76 (3)	2.10 (3)	2.809 (3)	156 (3)

Symmetry codes: (vi) *x*+1, *y*, *z*; (viii) *x*+1/2, −*y*+3/2, −*z*+1.

## References

[bb1] Altomare, A., Cascarano, G., Giacovazzo, C., Guagliardi, A., Burla, M. C., Polidori, G. & Camalli, M. (1994). *J. Appl. Cryst.* **27**, 435.

[bb2] Betteridge, P. W., Carruthers, J. R., Cooper, R. I., Prout, K. & Watkin, D. J. (2003). *J. Appl. Cryst.* **36**, 1487.

[bb3] Coppens, P. (1970). *Crystallographic Computing*, edited by F. R. Ahmed, S. R. Hall & C. P. Huber, pp. 255–270. Copenhagen: Munksgaard.

[bb4] Johnson, C. K. (1976). *ORTEPII* Report ORNL-5138. Oak Ridge National Laboratory, Tennessee, USA.

[bb5] Mackay, S., Edwards, C., Henderson, A., Gilmore, C., Stewart, N., Shankland, K. & Donald, A. (2000). *maXus* University of Glasgow, Scotland, MacScience Co., Yokohama, Japan, and Nonius BV, Delft, The Netherlands.

[bb6] Mehta, G. & Roy, S. (2008). *Tetrahedron Lett.* **49**, 1458–1460.

[bb7] Molecular Structure Corporation (1997). *TEXSAN* MSC, The Woodlands, Texas, USA.

[bb8] Nonius (2001). *COLLECT* Nonius BV, Delft, The Netherlands.

[bb9] Otwinowski, Z. & Minor, W. (1997). *Methods in Enzymology*, Vol. 276, *Macromolecular Crystallography*, Part A, edited by C. W. Carter Jr & R. M. Sweet, pp. 307–326. New York: Academic Press.

[bb10] Pinkerton, D. M., Banwell, M. G. & Willis, A. C. (2009). *Org. Lett.* **11**, 4290–4293. 10.1021/ol9016657 19743818

[bb11] Porco, J. A. Jr, Su, S., Lei, X., Bardhan, S. & Rychnovsky, S. D. (2006). *Angew. Chem. Int. Ed.* **45**, 5790–5792. 10.1002/anie.200602854 16871643

[bb12] Rychnovsky, S. D. (2006). *Org. Lett.* **8**, 2895–2898. 10.1021/ol0611346 16774284

[bb13] Schlegel, B., Härtl, A., Dahse, H.-M., Gollmick, F. A., Gräfe, U., Dörfelt, H. & Kappes, B. (2002). *J. Antibiot.* **55**, 814–817. 10.7164/antibiotics.55.814 12458771

